# Biventricular Pacemaker Implantation via the Femoral Vein

**DOI:** 10.4021/jocmr920w

**Published:** 2012-07-20

**Authors:** Sergio Agosti, Claudio Brunelli, Giovanni Bertero

**Affiliations:** a Department of Cardiology, University of Genoa, San Martino Hospital, Genoa, Italy

**Keywords:** Biventricular pacing, Cardiac resynchronization therapy, Femoral vein

## Abstract

We report the case of biventricular pacemaker implantation via the femoral vein, in a patient with impossibility of using standard superior vein approach and a contraindication to epicardial lead placement.

## Introduction

We report the case of a patient with inaccessible subclavian route and a contraindication to epicardial lead placement, in whom a biventricular pacemaker implantation was performed successfully via the femoral approach.

## Case Report

A 68-year-old male patient was admitted to our department with a diagnosis of idiopathic dilated cardiomyopathy (normal coronary arteries) associated with left bundle branch block (QRS duration of 140 msec) and reduced ejection fraction (EF, 35%).

In February 2004, a biventricular defibrillator was implanted in primary prevention. The patient responded well to treatment, showing significant clinical improvement (EF 45%) without experiencing arrhythmia episodes.

**Figure 1:**
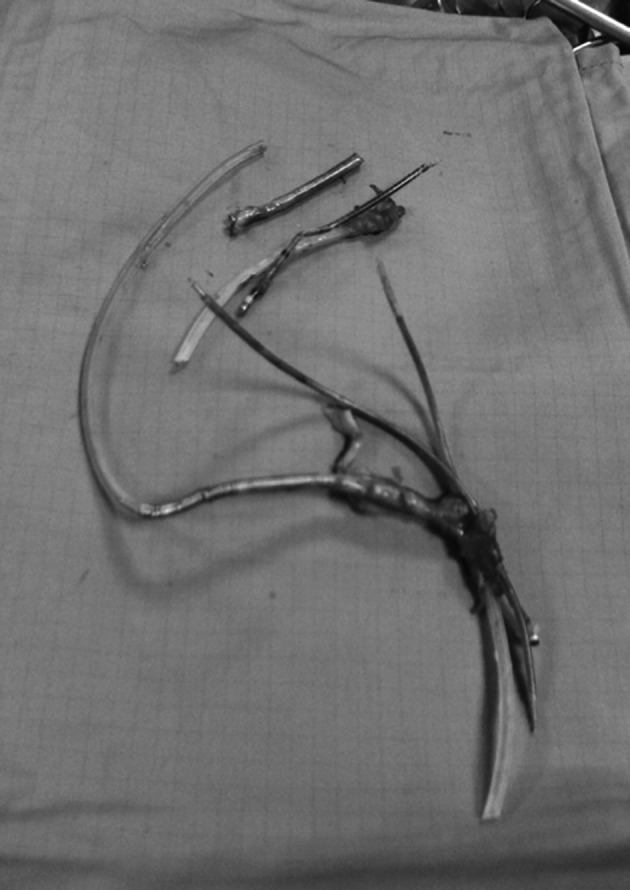
Leads removed during the surgical procedure.

In May 2009, the generator was replaced because of battery depletion. Six months later, displacement of the pulse generator to the subclavian vein and tunneling of the leads with subsequent pocket infection were observed. After a first attempt of percutaneous removal of the system with unsuccessful lead extraction, in February 2011 the patient underwent surgical lead removal (Fig. 1) and associated De Vega tricuspid annuloplasty. On that occasion, we decided not to implant a left ventricular epicardial lead in order to attempt transvenous lead placement at a later stage.

Unfortunately, the postoperative course was complicated by a “superior vena cava syndrome” with massive thrombosis of the innominate, internal jugular and subclavian veins. After a short stay in the hospital Rehabilitation Unit, the patient’s hemodynamic status deteriorated (NYHA class IV, EF 28%) and subsequent CRT was necessary. We considered the possibility of implanting a biventricular pacemaker to avoid epicardial lead placement because of the previous cardiac surgery procedure. However, phlebography showed complete occlusion of the upper extremity venous access and the femoral vein approach was used.

For easier lead placement and the likelihood that an optimal defibrillation vector could not be achieved at the level of the femoral vein, a biventricular pacemaker rather than a defibrillator was implanted. In addition, the patient was electrically stable and, in the past, responded well to CRT with a marked improvement in EF (> 35%).

### Procedure

During the procedure of biventricular pacemaker implantation via the femoral approach, three femoral venous accesses were obtained. The first catheter was placed into the left ventricle. A quadripolar Josephson catheter (TORQR series) was inserted into the coronary sinus (CS).

First, a peel-away delivery sheath (Attain Command 6250-MB2X, 50 cm in length) was introduced into the electrophysiology catheter. Owing to difficulties in cannulating the CS, it was replaced with a straight sheath (model 6257S, 57 cm in length), resulting in successful access to CS and good support.

**Figure 2:**
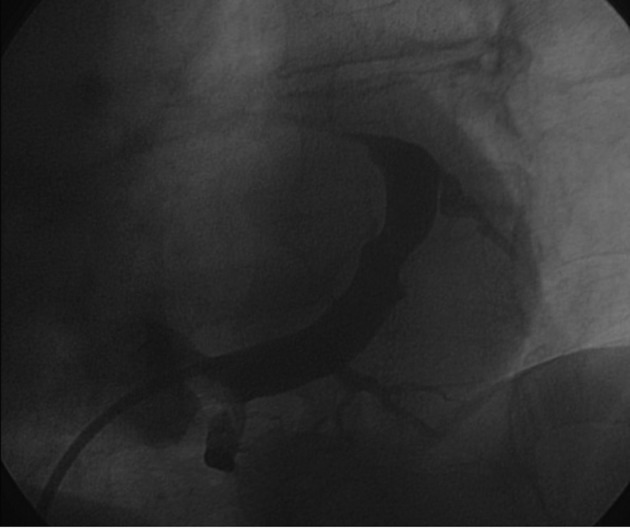
Sinogram showing target tributaries (30° left anterior oblique view).

Postero-lateral tributaries were visualized with non-occlusive CS venography (Fig. 2). A Medtronic Attain StarFix lead (model 4195, 103 cm in length) was inserted using the standard over-the-wire technique. The Attain StarFix lead is a 5 Fr, steroid-eluting, transvenous, unipolar, left ventricular, over the wire, cardiac vein pacing lead with three deployable lobes. During the implant procedure, the lobes can be undeployed by withdrawing the push tubing. Radiopaque markers assist in full lobe deployment. A threshold voltage of 2.5 V x 1.5 msec and a sensing threshold of 12 mV with good lead impedance were obtained. No diaphragmatic stimulation was noted at maximum output (10 V).

The active fixation mechanism of the Attain StarFix lead contributed to the success of the procedure, enabling easy removal of the delivery system.

**Figure 3:**
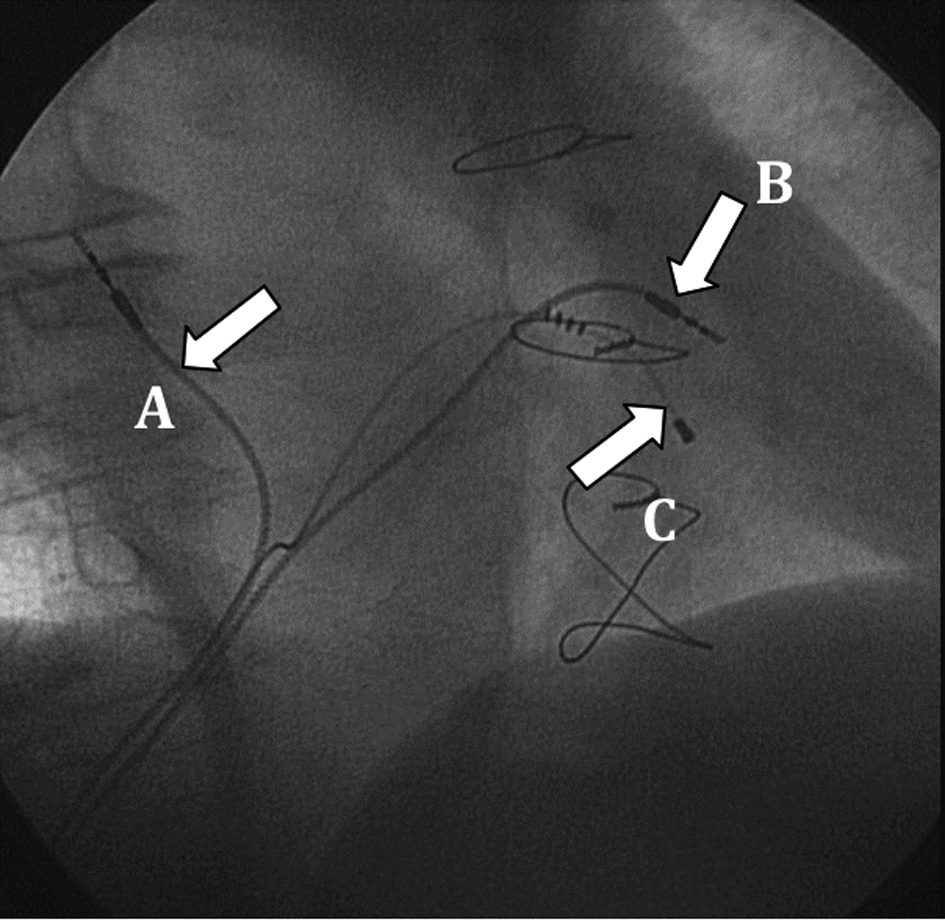
Final lead placement (30° right anterior oblique view): A: atrial lead; B: right ventricular lead; C: left ventricular lead.

The right ventricular lead was positioned in the high right ventricular septum, and the atrial lead was implanted in the right atrial septum. These locations allowed for an easier lead placement (Fig. 3). Both leads had active fixation (model 4076-80, 80 cm in length), with good voltage and sensing thresholds. The leads were tunneled through the rectus abdominis fascia and connected to the pulse generator in the abdominal pocket.

The patient was discharged after 4 days without complications. At pre-discharge follow-up, the left ventricular device had a voltage and sensing threshold of 2.5 V x 1.5 msec and 10 mV, respectively.

At one-month follow-up, the electrical measurements were stable with a voltage and sensing threshold of 1.0 V x 1.0 msec and 11.2 mV, respectively. The patient showed clinical improvement, also confirmed at echocardiography (EF 40%), suggesting a positive response to CRT.

At 1-year follow-up, the clinical and instrumental data remained stable with marked improvement in EF (45%).

## Discussion

Permanent pacemaker implantation via the femoral vein was first described by Ellestad et al in 1980 [1], and proved to be a viable alternative for patients with contraindications or impossibility of using standard superior vein approach. This technique also avoids unnecessary risk associated with thoracotomy and epicardial lead placement [2].

The reported indications for pacemaker implantation via the femoral vein include (i) obstruction of the subclavian vein or the superior vena cava; (ii) lead infections; (iii) mastectomy and/or thoracic radiotherapy; (iv) multiple leads in the superior vena cava; and (v) recurrent pocket erosion.

Despite being a safe approach with no risk of pneumothorax, this implantation technique is not widely adopted because it is associated with a high rate of lead dislodgment (up to 20%) [3], even when using active fixation catheters. In addition, the risk of complications with the femoral approach is not negligible.

Most available data on femoral pacing derive from series using temporary femoral pacing. These have shown it to be a safe procedure with low rates of thrombophlebitis [4].

The femoral approach for conventional pacemaker implantation is well described in the literature, but lead placement into the CS for CRT has been described only twice, using a passive [5] and active fixation lead [6], respectively.

Passive fixation leads, however, are more prone to dislodgment, whereas improved success rates of CRT implant may be achieved by employing the active fixation StarFix lead, specifically designed to reduce the risk of dislodgment.

Our experience confirms the feasibility of biventricular pacing through femoral vein access, facilitated by the use of an active fixation catheter. Most importantly, this technique allows to avoid surgical epicardial lead placement. The femoral approach should therefore be considered when deemed necessary and/or the superior venous route is inaccessible or contraindicated. This approach may prove a viable alternative to surgical epicardial lead placement.
